# Racial ideology, system justification, and just world belief in African Americans

**DOI:** 10.3389/fpsyg.2023.1193278

**Published:** 2023-12-08

**Authors:** Sydney Katherine Johnson, Kendra Thomas

**Affiliations:** ^1^Department of Educational Psychology, Ball State University, Muncie, IN, United States; ^2^Hope College, Holland, MI, United States

**Keywords:** racial ideology, justice, system justification, racial identity, fairness

## Abstract

Just world belief and system justification have previously been proposed to explain actions and beliefs of disadvantaged groups, but rarely together and never simultaneously in participants of color. A necessary expansion of work in this area is among African-American participants with differing views of race and how those views influence justice perceptions. Racial ideologies, used in African-American racial identity research, were studied as possible predictors of belief in a just world and system justification scores. The four racial ideologies examined in this study are assimilationist, humanist, nationalist, and oppressed minority. The current study examines belief in a just world and system justification as predicted by racial ideology. Participants (*n* = 261) responded to an online survey containing racial ideology items from the Multidimensional Model of Racial Identity (MMRI), the General and Personal Just World Scales, and the General System Justification Survey. Hierarchical linear regression was conducted, finding that nationalist ideology significantly predicted system justification and general just world belief.

## Introduction

African Americans, specifically those who descended from chattel slavery survivors, differ from other ethnic groups in the United States in that their identity is more closely tied to race than a cultural identity due to their unique history (Sellers et al., 1998). Even when comparing African Americans to other people of African descent living as minorities in other countries, African Americans differ in that their identity as African is not connected to a national, cultural, or tribal identity but instead is related to their race (Sellers et al., 1998; Hirsch, 2018). With African identity through nationality, cultural identity, or tribal identity come traditions and practices that not only promote a sense of self but also build intrapersonal connectedness in communities in ways that differ from the experiences of Black Americans (Hirsch, 2018). Instead, African-American culture and, similarly, identity stemming from being a Black American are heavily influenced by a combination of an understanding of America’s history of discrimination toward Black Americans and a very personal understanding of what this history means in the present for the individual (Du Bois, 1903; Cross, 1991).

Famously, the experiences of Black Americans have been captured in a number of works, such as, but not limited to, *The Souls of Black Folks* (Du Bois, 1903) and the essays and speeches of James Baldwin. In *The Souls of Black Folks*, Du Bois documented Black American experiences in the reconstruction era following the end of the American Civil War. In the text, Du Bois introduces the concept of double consciousness relating to the Black experience in America (Du Bois, 1903). With double consciousness, Du Bois highlighted a split in the minds of Black Americans as each person was forced to view themselves as a Black person in a white-dominated society during a time of racial terror and discrimination.

Baldwin discusses similar notions in many of his writings and speeches, with debatably his most famous study being *The Fire Next Time*. In the book, Baldwin discusses his experiences of discrimination growing up in Harlem in the 1930s and 1940s, as well as how racial discrimination eventually led to Baldwin’s decision to leave the United States for France in hopes of a better life (Baldwin, 1990).

This personal understanding of how America’s past and present relationship with prejudice and discrimination affects the individual helps shape African-American racial identity. While racial identity is not the only identity formation a Black person experiences, it can be present to differing degrees based on the narratives surrounding race that the individual has come to accept (Witherspoon et al., 2022). Equally important is how the individual attributes their race to being a part of their identity. There are a nearly innumerable number of books that historically cover African-American history in the United States; however, these personal accounts allow for an accounting of the formation of racial ideology. These personal accounts have yet to be examined in the psychological sciences in conjunction with system justification and just world belief. Researchers have previously captured these complexities of racial identity, creating the Multidimensional Model of Racial Identity (MMRI; Sellers et al., 1998). The MMRI allows researchers to study Black participants, going beyond ethnic identification and instead factoring in racial ideological beliefs and racial centrality, or how important race is to the individual’s identity (Sellers et al., 1998).

As previously mentioned, the unique position of African Americans, having a culture that is heavily influenced by experiences and effects of discrimination in American systems, may in turn influence beliefs about justice and fairness in systems and institutions.

Just world belief research has predominantly studied majority-white participants (Lerner, 1980; Lipkus et al., 1996; Dalbert, 1999; Correia and Dalbert, 2007; Thomas and Mucherah, 2018; Thomas and Rodrigues, 2019). Expanding research to include Black Americans allows researchers to begin to understand how societal positioning perspectives influence just world belief. Previous studies have also evaluated just world belief and system justification, but few have examined the constructs together with race being evaluated beyond demographic identification (Shockley et al., 2016). Evaluating just world belief, system justification, and racial ideology together provides information differentiating race from a basic demographic marker, personal perceptions of race in relation to justice beliefs, and congruent thinking between just world beliefs and system justification.

### Belief in a just world

Just world theory is often attributed to Melvin Lerner’s early experiments (Lerner, 1980). Participants were found to view people who received positive outcomes as more deserving and those who received negative outcomes as more culpable for their own misfortune (Lerner, 1965). However, Lerner’s early theory measured perceptions of justice and fairness for others, what is now referred to as general just world belief (G-BJW). Previous studies have found G-BJW to be associated with trust in authority figures and systems (Lerner, 1980). Belief in a just world research has expanded to examine personal just world belief (P-BJW), or perceptions of justice and fairness in the participant’s own life (Dalbert, 1999). P-BJW has previously been linked to greater wellbeing scores and higher self-esteem (Lipkus et al., 1996; Dalbert, 1999; Correia and Dalbert, 2007). It is believed that P-BJW serves as a kind of coping mechanism to act as a barrier in the individual’s mind from the injustices of the world (Dalbert, 1999; Hafer et al., 2019).

### System justification

Early system justification (SJ) research was influenced by the general belief in a just world theory. Its purpose was to understand why populations most dependent on systems and institutions are so likely to justify or ignore misbehavior (Jost and Banaji, 1994). These initial studies found SJ to be associated with higher beliefs in meritocratic legislation, as well as right-wing authoritarianism, social dominance orientation, and a Protestant work ethic (Jost, 2019). Research in this construct among African Americans has indicated that African Americans from lower socioeconomic backgrounds were more likely to record greater SJ scores (Jost et al., 2003) However, other studies have examined the rationale used to justify systems, with some researchers speculating that individuals that feel closer to majority groups are more likely to try to find ways to justify the exclusivity of their fortune (Caricati and Owuamalam, 2020). In African-American populations, it is possible that differences in the adoption of these rationales could affect system-justifying beliefs (Shockley et al., 2016). The differing results suggest that there need to be more studies conducted that not only allow for further examination but also more precise measurements of how beliefs about belonging to a minority group shape perceptions of systems and institutions.

### Racial ideology

In the 1990s, calls for a racial identity measure specifically for African Americans led to the creation of the Multidimensional Model of Racial Identity, which measures four racial ideologies: assimilationist, humanist, nationalist, and oppressed minority, as well as other concepts such as racial centrality (Rowley et al., 1998; Sellers et al., 1998). The MMRI has since been adapted as the Multidimensional Inventory of Black Identity (MIBI), as components of the model are still in use in African-American racial identity research (Neblett Jr et al., 2013; Cooper et al., 2019).

Racial centrality is best defined as how central race is to an individual’s identity (Sellers et al., 1998). Assimilationist ideology represents the idea that wellbeing is best achieved by succeeding within existing social, financial, and professional institutions (Sellers et al., 1998). Humanist ideology suggests that individuals value individualism and, while conscious of their race, place their personhood as more relevant to their identity (Sellers et al., 1998). Next, the oppressed minority ideology centers on the belief that the plight of African Americans in the United States is similar and connected to the discrimination of other disenfranchised groups (Sellers et al., 1998). Finally, nationalist ideology centers on the uniqueness of the oppression of the African-American community and is more likely to hold separatist views (Sellers et al., 1998).

Each construct was originally designed to come together within the larger model to form a racial identity for Black Americans (Sellers et al., 1998). However, the MIBI is often used for individual components instead of the full racial identity measure (Yip et al., 2006; Leath and Chavous, 2017). This is also partially due to intercorrelation between constructs within the measure, leading to multicollinearity during data analysis (Vandiver et al., 2009).

The MIBI has previously been used in studies investigating African-American academic attainment and civil engagement (Chavous et al., 2003; Leath and Chavous, 2017), but few studies exist that couple racial identity in African Americans with just world belief. Even fewer studies exist that go further, adding system justification. Each ideology with its respective definitions highlights a different conception of race and, therefore, should be examined as a predictor of SJ and BJW.

### Study aims

This study aimed to examine the predictive relationships between the dimensions of racial ideology (assimilationist, humanist, nationalist, and oppressed minority) and personal just world belief (P-BJW), general just world belief (G-BJW), and system justification (SJ) among African Americans. Assimilationist and humanist ideologies were hypothesized to predict positive P-BJW, G-BJW, and SJ scores. Nationalist ideology was expected to negatively predict P-BJW, G-BJW, and SJ scores. Finally, oppressed minority ideology was expected to negatively predict P-BJW, G-BJW, and SJ scores. High racial centrality scores are expected to amplify negative scores and positive scores in P-BJW and G-BJW, respectively.

## Methods

### Procedure

In this cross-sectional study, an online survey was disseminated to African-American participants. Upon choosing to participate in the survey, participants were presented with informed consent information. After providing consent, participants electronically responded to the survey. Ethical approval was provided by the institutional review board of the University of Indianapolis (IRB number: 10287).

### Participants and recruitment

The inclusion criteria for participation in the current study were identifying as an African American and being over the age of 18. Originally, participants were to be recruited solely via Survey Monkey, published on Facebook and Instagram. However, due to a low response rate, the survey was moved from Survey Monkey and the aforementioned social media platforms to Cloud Research. Cloud Research is a service provided by Amazon that connects researchers to participants who will be compensated for their responses to surveys they chose to respond to.

In this study, participants who responded through Cloud Research were compensated for their contributions by $0.50, while participants recruited through social media were not compensated. Participants were presented with informed consent information before starting the survey. All data collection was anonymous. Data collection was conducted from June to September of 2020.

### Measures

The online survey contained 71 items, including sociodemographics, P-BJW, G-BJW, racial ideology, racial centrality, and SJ.

#### Socio-demographics

Participants were first asked to answer four demographic questions regarding age, gender, highest completed education level, and race, out of a desire to confirm race before beginning the survey, as identifying as African American was a requirement for participating in the study.

#### Racial ideology and racial centrality

Items were drawn from the Multidimensional Model of Racial Identity (Sellers et al., 1998). Racial ideology items were divided into four groups: assimilationist (e.g., “A sign of progress is that Blacks are in the mainstream of America more than ever before.,” 𝛼 = 0.77), humanist (e.g., “We are all children of a higher being, therefore, we should love people of all races.,” 𝛼 = 0.81), nationalist (e.g., “Blacks would be better off if they adopted Afrocentric values.,” 𝛼 = 0.74), and oppressed minority (e.g., “Blacks should learn about the oppression of other groups.,” 𝛼 = 0.82). All eight racial centrality items (e.g., “Being Black is an important reflection of who I am.,” 𝛼 = 0.84) were included. Items were scored on a seven-point Likert scale ranging from “strongly disagree to strongly agree.” Items for each ideology were averaged for the final scores. Higher scores indicate that the participant agrees with the ideology, while lower scores indicate disagreement.

#### General and personal just world belief

The general and personal just world scales contain 13 items (Dalbert, 1999). The first six items provided examined general items (e.g., “I think the world is basically a just place,” 𝛼 = 0.66) followed by seven personal items (e.g., “I am usually treated fairly,” 𝛼 = 0.86). Items from both scales were scored on a 7-point Likert scale and averaged for a final score. A higher score in G-BJW means the participant feels the world is more just, whereas a low score indicates a belief that the world is not fair. A higher score in P-BJW means a higher belief that their personal world is fair, while a low score indicates a belief that their world is not fair.

#### System justification

The general just world survey (Roccato et al., 2014) was used. It consists of eight items asking about trust in government and belief in societal fairness (e.g., “In general, you find society to be fair,” 𝛼=0.85). Items were scored on a 7-point Likert scale with two items (“American society needs to be radically restructured” and “Our society is getting worse every year”) reverse coded. Final scores were determined by collecting the average between the eight items. A higher score means that participants believe systems are trustworthy and are more likely to justify the system, while a low score means low system trust.

### Data analysis

To measure the effects of racial ideology on system justification and just world beliefs, results were analyzed as hierarchical regressions, expecting to see predictive relationships. Data collected from Cloud Research and Survey Monkey were analyzed together due to the low numbers recruited through Survey Monkey. To further examine differences between the sample types, *t*-tests were conducted on all of the variables, and there were significant differences in G-BJW, P-BJW, and SJ.

## Results

In total, 261 participants completed the online survey. A total of 36 participants were recruited through social media platforms, and 225 participants responded to the survey through Cloud Research’s services. A total of 32 responses were removed for not meeting the initial requirements of identifying as African American and being above 18 years of age to participate.

Of the 229 participants that remained in the data pool, 62.7% identified as female and 37.3% identified as male, with a range of 18 to 79 years old (*M* = 35.11, SD = 12.88). Respondents recruited through Cloud Research on average attended some college or completed an associate’s degree, while the participants recruited through social media on average completed a bachelor’s degree, with a range of less than high school to doctorate for all participants. Some college attendance accounted for 30.1% of total recruits, followed by high school graduates (25.3%), 4-year degrees (20.1%), 2-year degrees (15.7%), and professional degrees (7.9%). One participant reported less than high school as their highest completed level (0.4%), and one participant reported receiving a doctoral degree (0.4%). Educational attainment was collected to serve as a proxy for socioeconomic status. Both data sets were analyzed together due to the small number of participants recruited through social media.

After an independent sample *t*-test, the statistical differences between the two groups were confirmed to be statistically significant among all three dependent variables. Though similar in age and gender, the social media sample was found to be significantly different in racial ideology scores, racial centrality scores, SJ scores, and G-BJW scores. The average SJ score for Cloud Research participants was significantly higher than the sample recruited through social media. Racial centrality was significantly higher in the social media sample, and G-BJW scores were significantly lower in the social media group (see Table 1).

**Table 1:** The *t*-test results comparing recruitment methods on ideological leanings, SJ, G-BJW, and P-BJW.

	Social media	Cloud research	*t*
	M (SD)	M (SD)	
Assimilationist	4.53 (1.05)	3.43 (1.07)	−5.569^***^
Humanist	4.83(0.71)	3.36 (1.17)	−9.969^***^
Nationalist	4.63(0.89)	3.97 (1.18)	−3.795^***^
Oppressed Minority	3.61 (1.07)	4.70(0.99)	−5.888^***^
Racial centrality	5.89(0.90)	3.48 (1.06)	−13.982^***^
SJ	2.25(0.66)	4.55 (1.01)	12.748^***^
G-BJW	3.09 (1.01)	4.37(0.99)	6.844^***^
P-BJW	3.82 (1.19)	4.00 (1.19)	0.801

^***^p < 0.001. M, mean; SD, standard deviation. All variables range 1 (strongly disagree) to 7 (strongly agree).

Humanist ideology was the only ideology found to be correlated with education (*p* < 0.01, r = 0.132). Racial centrality was positively correlated with assimilationist (*p* < 0.001, r = 0.218), nationalist (*p* < 0.001, r = 0.386) and oppressed minority (*p* < 0.001, r = 0.315) ideologies but negatively correlated with humanist ideology (*p* < 0.001, r = −0.455). G-BJW and P-BJW were found to be positively correlated (*p* < 0.001, r = 0.516). Racial centrality and SJ were found to be negatively correlated (*p* < 0.001, r = −0.143; see Supplementary material).

Racial centrality was the strongest predictor of system justification scores with a significant negative relationship (𝛽 = −0.50, *p* < 0.001), followed by humanist ideology (β = −0.17, *p* < 0.05), which was also negatively predictive of system justification. Assimilationist and nationalist scores were both found to be positively predictive of SJ (β = 0.26, *p* < 0.01; β = 0.13, *p* < 0.05; see Table 2).

**Table 2:** Regression analysis of racial ideology predicting system justification.

	*B*	*SE B*	*β*
Assimilationist	0.294	0.106	0.259^**^
Humanist	−0.178	0.089	−0.173^*^
Nationalist	0.146	0.071	0.133^*^
Oppressed minority	−0.115	0.102	−0.102
Racial centrality	−0.467	0.061	−0.496^***^
F		14.857	
*R* ^2^		0.252	

^*^p < 0.05, ^**^p < 0.01, ^***^p < 0.001.

Nationalist ideology was the only ideology found to be a significant positive predictor of G-BJW (β = 0.23, *p* < 0.01). Racial centrality was found to be significantly negatively predictive of G-BJW (β = −0.39, *p* < 0.01), indicating that those who hold their race as an important part of their identity are less likely to believe the world is just. See Table 3 for all the results of these analyses.

**Table 3:** Regression analysis of racial ideology predicting G-BJW.

	*B*	*SE B*	*β*
Assimilationist	0.118	0.096	0.122
Humanist	0.01	0.081	0.011
Nationalist	0.211	0.064	0.227^**^
Oppressed minority	−0.016	0.093	−0.017
Racial centrality	−0.315	0.056	−0.392^**^
F		7.179	
*R* ^2^		0.14	

^*^p < 0.05, ^**^p < 0.01, ^***^p < 0.001.

Assimilationist and nationalist ideologies significantly predicted positive P-BJW scores (β = 0.31, *p* < 0.01; β = 0.22, *p* < 0.01). Humanist, oppressed minority, and racial centrality scores were found to have no significant relationship (Table 4).

**Table 4:** Regression analysis of racial ideology predicting P-BJW.

	*B*	*SE B*	*β*
Assimilationist	0.328	0.104	0.314^**^
Humanist	0.098	0.088	0.103
Nationalist	0.219	0.07	0.218^**^
Oppressed minority	−0.124	0.101	−0.119
Racial centrality	−0.077	0.06	−0.088
F		7.638	
*R* ^2^		0.148	

^*^p < 0.05, ^**^p < 0.01, ^***s^*p* < 0.001.

## Discussion

The current study found racial centrality significantly predicted SJ, yielding a negative relationship. System justification was also found to be negatively predicted by humanist ideology and positively predicted by assimilationist and nationalist ideology. This study’s results indicating that assimilationist and nationalist ideology were predictive of SJ are reflective of early studies that found African Americans likely to justify systems but provide a needed caveat (Jost and Banaji, 1994). Humanist ideology negatively predicting SJ did not support previous research as system justification was believed to be broadly predicted by belonging to a disadvantaged group, as well as if the participant ascribed to other ideologies (Jost and Banaji, 1994; Jost et al., 2004). The negative relationship between racial centrality and SJ contradicts previous research as well (Jost et al., 2004). Instead, results from this study indicate it is possible certain ideological frameworks are over-represented in research, leading to ethnicities being represented by participants holding ideological beliefs that positively predict SJ.

Racial centrality negatively predicted G-BJW. Contrary to the hypothesis, G-BJW was also positively predicted by nationalist ideology. Nationalist ideology positively predicting G-BJW indicates first that G-BJW for people who feel most vulnerable to injustice is a means of hope and order (Lerner, 1980). Second, nationalist ideology positively predicting G-BJW indicates that G-BJW is a different construct than SJ and that, when studied together, the constructs can provide clarity as to how participants view organizations, institutions, government, and other large entities outside of themselves. Assimilationist ideology was found to have no statistically significant relationship with G-BJW. The distinction in predictors suggests that G-BJW and SJ as constructs measure two different things, as they should, and that investigating G-BJW and SJ as unique constructs in the same study is justified.

Given this theoretical history with SJ being theoretically derived from BJW (Jost and Banaji, 1994), it is sensible to see whether one ideology could predict both G-BJW and SJ as nationalist ideology did in this study. However, the analysis of assimilationist ideology and finding a statistically significant positive relationship with SJ and not G-BJW suggests a recognition that the world may not be fair or just, but a belief that systems and institutions operate in some form of fairness and justice.

P-BJW scores were positively predicted by assimilationist and nationalist ideologies. While P-BJW being positively predicted by assimilationist ideology was hypothesized, humanist ideology was expected to have a positive association as well, while nationalist ideology was expected to be negatively related. It should be noted that conceptually, assimilationist and nationalist ideologies are almost opposites, with one finding solace within predominantly white institutions in terms of bettering African Americans, while the other esteems unity among African Americans and, in some cases, separatism, believing African Americans alone should be in places of power in institutions that predominantly serve other African Americans (Sellers et al., 1998). While there is no evidence to point to what exactly causes this, results from this study suggest that assimilationist and nationalist ideologies may provide a kind of coping mechanism when faced with injustice, given the benefits of higher P-BJW (Lipkus et al., 1996; Dalbert and Filke, 2007).

## Limitations and future directions

At the beginning of this study, recruitment was done exclusively through social media, which would allow for responses from participants who may feel skeptical about how research is conducted. This method led to poor engagement, which led to the use of Cloud Research for the completion of the survey. When making comparisons between the two groups, there were notable differences, as described in the Results section, finding significant differences in demographic data as well as in scores in each of the examined constructs in the study.

Prior to data analysis, a decision was made to analyze the Survey Monkey participants with the data from the Cloud Research sample. This decision was made on the belief that the sample recruited through Survey Monkey may access a population of Black Americans that may not be accounted for in psychological research. Nevertheless, it should be noted that while the additional participants increase the statistical power of the study, it is possible that the Survey Monkey sample is simply an outlier affecting the study itself. Future research is needed to confirm or refute the findings of this study, especially as it pertains to differences between the two samples included in this study.

The study overall does have a relatively small sample size (*N* = 261), preventing any generalizations about the study’s findings. However, the study does provide evidence to suggest that African-American racial ideology may be predictive of just world and system justification beliefs. As just world belief and system justification have both been theorized to provide psychological protection from uncertainty and feelings of injustice, this study should be examined further to determine if system justification and just world beliefs can be especially harmful to those that ascribe to different racial ideologies.

## Conclusion

This study evaluated if African-American racial ideologies influenced just world beliefs and system justification responses. The results of this study indicated that African-American racial centrality was predictive of G-BJW and SJ beliefs, with P-BJW being predicted by assimilationist and nationalist views. Racial centrality and racial ideology provided an extra lens through which the relationship between race, justice beliefs, and system justification could be examined.

## Data availability statement

The raw data supporting the conclusions of this article will be made available by the authors, without undue reservation.

## Ethics statement

The studies involving humans were approved by University of Indianapolis Institutional Review Board. The studies were conducted in accordance with the local legislation and institutional requirements. The participants provided their written informed consent to participate in this study.

## Author contributions

SJ and KT contributed to the writing and editing of the manuscript as well as statistical analyses. All authors contributed to the article and approved the submitted version.
