# Supplementary material for: Racial ideology, system justification, and just world belief in African Americans
Source: Front Psychol. 2023 Dec 8;14:1193278. doi: 10.3389/fpsyg.2023.1193278 (PMC10739316; doi:10.3389/fpsyg.2023.1193278)
Supplement: Supplementary file 1 [file Data_Sheet_1.PDF]

Correlations of variables.

	1	2	3	4	5	6	7	8
1. Education	-							
2. Assimilationist	0.032	-						
3. Humanist	.132*	.705**	-					
4. Nationalist	0.102	.203**	-0.072	-				
5. Oppressed Minority	0.078	.702**	-.143*	.301**	-			
6. Racial Centrality	0.121	.218**	-.455**	.386**	.315**	-		
7. G-BJW	-.219**	0.083	0.044	0.106	0.035	-.278**	-	
8. P-BJW	-.151*	.304**	.271**	.226**	.223**	0.06	.516**	-
9. SJ	-.229**	-0.027	-.149*	-0.072	-.143*	-.455**	.499**	.414**

Note. * $p<.01$, ** $p<.001$
